# Supplementary material for: At Least Seven Distinct Rotavirus Genotype Constellations in Bats with Evidence of Reassortment and Zoonotic Transmissions
Source: mBio. 2021 Jan 19;12(1):e02755-20. doi: 10.1128/mBio.02755-20 (PMC7845630; doi:10.1128/mBio.02755-20)
Supplement: TABLE S1 [file mBio.02755-20-st001.docx]

**Table S1.** RT-PCR oligonucleotides for the initial rotavirus screening against VP1

| **ID no.** | **Sequence (5’ → 3’)** | **Position** | **Genome segment** | **Polarity** | **Assay type** |
| --- | --- | --- | --- | --- | --- |
| PanRota-F1570 | TAYACIGAYGTITCICARTGGGA | 1570-1593^a^ | VP1 | + | Heminested RT-PCR, 1^st^ round |
| PanRota-R1922 | GCGTAGTTGTCGTCICCRTCBAC | 1900-1922^a^ | VP1 | - | 1^st^ and 2^nd^ rd |
| PanRota-F1585a | CARTGGGATTCGTCICAGCAYAAYAC | 1585-1610^a^ | VP1 | + | 2^nd^ rd |
| PanRota-F1585b | CARTGGGACGCCAGICAACATAAYAC | 1585-1610^a^ | VP1 | + | 2^nd^ rd |

ID, identification; RT-PCR, reverse transcription–PCR; ^a^corresponding to Rotavirus A G11P[25] Dhaka6 VP1 (GenBank # EF560705); Variant forms of primers (marked consecutively with an alphabetic character in the last position) were mixed together equally and from then on treated as one single primer.
