# Supplementary material for: At Least Seven Distinct Rotavirus Genotype Constellations in Bats with Evidence of Reassortment and Zoonotic Transmissions
Source: mBio. 2021 Jan 19;12(1):e02755-20. doi: 10.1128/mBio.02755-20 (PMC7845630; doi:10.1128/mBio.02755-20)
Supplement: TABLE S2 [file mBio.02755-20-st002.docx]

**Table S2.** Taxonomical annotation, sampling time and location, RVA PCR detection information of the bat samples

| **Order-Family** | **Species** | **No. of samples**  **per sampling site and year** | | | | | | | | **PCR positive (%)** | **Positive samples (ID)** |
| --- | --- | --- | --- | --- | --- | --- | --- | --- | --- | --- | --- |
|  |  | **total** | **BGR 2008** | **BGR 2009** | **CRC**  **2010** | **GAB 2009** | **DEU 2008** | **GHA 2009** | **ROU 2008** |  |  |
| **Chiroptera-Pteropodidae** | *Eidolon helvum* | **226** |  |  |  |  |  | 226 |  | 1 (0.4%) | K212 |
|  | *Micropteropus pusillus* | **1** |  |  |  |  |  | 1 |  | 0 (0%) |  |
|  | *Rousettus aegyptiacus* | **10** |  |  |  | 8 |  | 2 |  | 0 (0%) |  |
| **Chiroptera-Rhinolophidae** | *Rhinolophus blasii* | **90** | 82 | 8 |  |  |  |  |  | 1 (1.1%) | BB89-15 |
|  | *Rhinolophus euryale* | **336** | 244 | 92 |  |  |  |  |  | 2 (0.6%) | BBR89-2, BR89-60 |
|  | *Rhinolophus ferrum-equinum* | **52** | 45 | 6 |  |  |  |  | 1 | 0 (0%) |  |
|  | *Rhinolophus hipposideros* | **6** | 6 |  |  |  |  |  |  |  |  |
|  | *Rhinolophus landeri* | **1** |  |  |  |  |  | 1 |  | 0 (0%) |  |
|  | *Rhinolophus mehelyi* | **22** | 14 | 8 |  |  |  |  |  | 0 (0%) |  |
|  | *Rhinolophus spec.* | **6** |  |  |  | 6 |  |  |  | 0 (0%) |  |
| **Chiroptera-Hipposideridae** | *Hipposideros cf ruber/caffer* | **183** |  |  |  | 46 |  | 137 |  | 2 (1.1%) | GKS-637, GKS-660 |
|  | *Hipposideros cf spec* | **2** |  |  |  |  |  | 2 |  | 0 (0%) |  |
|  | *Macronycteris*  *gigas* | **67** |  |  |  | 67 |  |  |  | 10 (14.9%) | GKS-897, GKS-912, GKS-926, GKS-929, GKS-934, GKS-941, GKS-942, GKS-953, GKS-954, GKS-955 |
|  | *Hipposideros abae* | **62** |  |  |  |  |  | 62 |  | 0 (0%) |  |
| **Chiroptera-Nycteridae** | *Nycteris spec.* | **3** |  |  |  |  |  | 3 |  | 0 (0%) |  |
| **Chiroptera-Emballonuridae** | *Coleura afra* | **5** |  |  |  |  |  | 5 |  | 0 (0%) |  |
|  | *Peropteryx kappleri* | **5** |  |  | 5 |  |  |  |  | 0 (0%) |  |
| **Chiroptera-Phyllostomidae** | *Anoura geoffroyi* | **100** |  |  | 100 |  |  |  |  | 0 (0%) |  |
|  | *Carollia castanea* | **1** |  |  | 1 |  |  |  |  | 0 (0%) |  |
|  | *Carollia perspicillata* | **203** |  |  | 203 |  |  |  |  | 1 (0.5 %) | KCR10-93 |
|  | *Enchisthenes hartii* | **3** |  |  | 3 |  |  |  |  | 0 (0%) |  |
|  | *Glossophaga commissarisi* | **3** |  |  | 3 |  |  |  |  | 0 (0%) |  |
|  | *Glossophaga soricina* | **22** |  |  | 22 |  |  |  |  | 0 (0%) |  |
| **Chiroptera-Mormoopidae** | *Pteronotus parnellii* | **21** |  |  | 21 |  |  |  |  | 0 (0%) |  |
| **Chiroptera-Natalidae** | *Natalus lanatus* | **3** |  |  | 3 |  |  |  |  | 0 (0%) |  |
| **Chiroptera-Vespertilionidae** | *Barbastella barbastellus* | **13** | 12 |  |  |  |  |  | 1 | 0 (0%) |  |
|  | *Miniopterus inflatus* | **2** |  |  |  | 2 |  |  |  | 0 (0%) |  |
|  | *Miniopterus schreibersii* | **77** | 39 |  |  |  |  |  | 38 | 0 (0%) |  |
|  | *Myotis brandtii* | **17** |  |  |  |  | 17 |  |  | 0 (0%) |  |
|  | *Myotis alcathoe* | **2** | 2 |  |  |  |  |  |  | 0 (0%) |  |
|  | *Myotis bechsteinii* | **57** | 32 |  |  |  | 25 |  |  | 0 (0%) |  |
|  | *Myotis capaccini* | **1** | 1 |  |  |  |  |  |  | 0 (0%) |  |
|  | *Myotis dasycneme* | **149** |  |  |  |  | 149 |  |  | 0 (0%) |  |
|  | *Myotis daubentonii* | **110** | 7 |  |  |  | 103 |  |  | 1 (0.9%) | SW78-39 |
|  | *Myotis emarginatus* | **5** | 5 |  |  |  |  |  |  | 0 (0%) |  |
|  | *Myotis myotis* | **77** | 3 |  |  |  | 60 |  | 14 | 0 (0%) |  |
|  | *Myotis mystacinus* | **51** |  |  |  |  | 51 |  |  | 0 (0%) |  |
|  | *Myotis nattereri* | **27** | 2 |  |  |  | 25 |  |  | 0 (0%) |  |
|  | *Myotis oxygnathus* | **22** | 1 |  |  |  |  |  | 21 | 0 (0%) |  |
|  | *Nyctalus leisleri* | **3** | 3 |  |  |  |  |  |  | 0 (0%) |  |
|  | *Nyctalus noctula* | **11** |  |  |  |  | 2 |  | 9 | 0 (0%) |  |
|  | *Pipistrellus cf nanus/nanulus* | **3** |  |  |  |  |  | 3 |  | 0 (0%) |  |
|  | *Pipistrellus nathusii* | **2** |  |  |  |  | 2 |  |  | 0 (0%) |  |
|  | *Pipistrellus pipistrellus* | **37** |  |  |  |  | 37 |  |  | 0 (0%) |  |
|  | *Pipistrellus pygmaeus* | **29** | 2 |  |  |  | 27 |  |  | 0 (0%) |  |
|  | *Pipistrellus spec.* | **6** |  |  |  |  |  | 6 |  | 0 (0%) |  |
|  | *Plecotus auritus* | **5** | 2 |  |  |  | 3 |  |  | 0 (0%) |  |
|  | *Plecotus austriacus* | **1** |  |  |  |  | 1 |  |  | 0 (0%) |  |
| **Chiroptera-Molossidae** | *Mops spec.* | **2** |  | 1 |  |  |  | 1 |  | 0 (0%) |  |
|  | **Total (46 species)** | **2142** | **502** | **115** | **361** | **129** | **502** | **449** | **84** | **18 (0.8%)** |  |

Country: BGR = Bulgaria; CRC = Costa Rica; GAB = Gabon; DEU = Germany; GHA = Ghana; ROU = Romania
