# Supplementary material for: At Least Seven Distinct Rotavirus Genotype Constellations in Bats with Evidence of Reassortment and Zoonotic Transmissions
Source: mBio. 2021 Jan 19;12(1):e02755-20. doi: 10.1128/mBio.02755-20 (PMC7845630; doi:10.1128/mBio.02755-20)
Supplement: TABLE S3 [file mBio.02755-20-st003.docx]

**Table S3.** RVA-positive bat samples detected by targeted RT-PCR and undergone viral metagenomics

| Sample | Host | Country | Place | Year |
| --- | --- | --- | --- | --- |
| BBR89-2 | *Rhinolophus euryale* | Bulgaria | Bratanova | 2008 |
| BB89-15 | *Rhinolophus blasii* |  | Elenas Cave |  |
| BR89-60 | *Rhinolophus euryale* |  | Roman Horse Cave |  |
| SW78-39 | *Myotis daubentonii* | Germany | Wahlstorf, SH | 2008 |
| GKS-660 | *Hipposideros caffer* | Gabon | Zadie | 2009 |
| GKS-637 |  |  |  |  |
| GKS-897 | *Macronycteris*  *gigas* | Gabon | Faucon | 2009 |
| GKS-912 |  |  |  |  |
| GKS-926 |  |  |  |  |
| GKS-929 |  |  |  |  |
| GKS-934 |  |  |  |  |
| GKS-941 |  |  |  |  |
| GKS-942 |  |  |  |  |
| GKS-953 |  |  |  |  |
| GKS-954 |  |  |  |  |
| GKS-955 |  |  |  |  |
| K212 | *Eidolon helvum* | Ghana | Kumasi | 2009 |
| KCR10-93 | *Carollia perspicillata* | Costa Rica | Orosi | 2010 |
