# Supplementary material for: At Least Seven Distinct Rotavirus Genotype Constellations in Bats with Evidence of Reassortment and Zoonotic Transmissions
Source: mBio. 2021 Jan 19;12(1):e02755-20. doi: 10.1128/mBio.02755-20 (PMC7845630; doi:10.1128/mBio.02755-20)
Supplement: FIG S1 [file mBio.02755-20-sf001.docx]

**Figure S1.** RVA-positive bat families and species. The RVA-positive bat families reported in the present study (red) and in literature (blue) are shown on the phylogenetic tree adapted from Simmons et al (2003). No RVA is reported in families in black. A family is accepted positive for the literature group if more than 1 RVA segment was submitted to GenBank.
The corresponding bat species and the country of sample collection are also displayed. Country: GHA = Ghana, FRA = France, BRA = Brazil, ZMB = Zambia, SAU = Saudi Arabia, CRC = Costa Rica, KEN = Kenya, CHN = China, BGR = Bulgaria, GAB = Gabon
