# Supplementary material for: At Least Seven Distinct Rotavirus Genotype Constellations in Bats with Evidence of Reassortment and Zoonotic Transmissions
Source: mBio. 2021 Jan 19;12(1):e02755-20. doi: 10.1128/mBio.02755-20 (PMC7845630; doi:10.1128/mBio.02755-20)
Supplement: TABLE S4 [file mBio.02755-20-st004.docx]

**Table S4**. a. Examples of reassortments among bat RVA strains, b. Examples of bat RVA strains with unusual genotype constellations, potentially resulting from (multiple) reassortment events, c. Examples of distinct RVA genotype constellations in the same bat species

| **a. Strains** | **VP7** | **VP4** | **VP6** | **VP1** | **VP2** | **VP3** | **NSP1** | **NSP2** | **NSP3** | **NSP4** | **NSP5** | **Host Species** | **Host Family** | **Diet** |
| --- | --- | --- | --- | --- | --- | --- | --- | --- | --- | --- | --- | --- | --- | --- |
| **RVA/Bat-wt/BGR/BB89-15/2008/G3P[3]** | G3 | P[3] | I3 | R3 | C3 | M3 | A9 | N3 | T3 | E3 | H6 | *Rhinolophus blasii* | *Rhinolophidae* | I |
| **RVA/Bat-wt/BGR/BR89-60/2008/G3P[3]** | G3 | P[3] | I3 | R3 | C3 | M3 | A9 | N3 | T3 | E3 | H6 | *Rhinolophus euryale* | *Rhinolophidae* | I |
| RVA/Bat-wt/CHN/LZHP2/2015/G3P[3] | G3 | P[3] | I3 | R3 | C3 | M3 | A9 | N3 | T3 | E3 | H6 | *Hipposideros pomona* | *Hipposideridae* | I |
| RVA/Bat-tc/CHN/MSLH14/2012/G3P[3] | G3 | P[3] | I8 | R3 | C3 | M3 | A9 | N3 | T3 | E3 | H6 | *Rhinolophus hipposideros* | *Rhinolophidae* | I |
| RVA/Bat-tc/CHN/MYAS33/2013/G3P[10] | G3 | P[10] | I8 | R3 | C3 | M3 | A9 | N3 | T3 | E3 | H6 | *Aselliscus stoliczkanus* | *Hipposideridae* | I |
| RVA/Bat-wt/CHN/BSTM70/2015/G3P[3] | G3 | P[3] | I8 | R3 | C3 | M3 | A29 | N3 | T3 | E3 | H6 | *Taphozous melanopogon* | *Emballonuridae* | I/F |
| RVA/Bat-wt/CHN/YSSK5/2015/G3P[3] | G3 | P[3] | I8 | R20 | C2 | M1 | A9 | N3 | T3 | E3 | H6 | *Scotophilus kuhlii* | *Vespertilionidae* | I |

| **b. Strains** | **VP7** | **VP4** | **VP6** | **VP1** | **VP2** | **VP3** | **NSP1** | **NSP2** | **NSP3** | **NSP4** | **NSP5** | **Host Species** | **Host Family** | **Diet** |
| --- | --- | --- | --- | --- | --- | --- | --- | --- | --- | --- | --- | --- | --- | --- |
| RVA/Bat-wt/ZMB/LUS12-14/2012/G3P[3] | G3 | P[3] | I3 | R2 | C2 | M3 | A9 | N2 | T3 | E2 | H3 | *Rhinolophus simulator* | Rhinolophidae | I |
| RVA/Bat-wt/CHN/YSSK5/2015/G3P[3] | G3 | P[3] | I8 | R20 | C2 | M1 | A9 | N3 | T3 | E3 | H6 | *Scotophilus kuhlii* | Vespertilionidae | I |
| RVA/Bat-wt/KEN/322/Kwale/2015/G3P[10] | G3 | P[10] | I2 | R8 | C3 | M5 | A5 | N3 | T6 | E3 | H6 | *Taphozous mauritianus* | Emballonuridae | I |

| **c. Strains** | **VP7** | **VP4** | **VP6** | **VP1** | **VP2** | **VP3** | **NSP1** | **NSP2** | **NSP3** | **NSP4** | **NSP5** | **Host Species** | **Host Family** | **Diet** |
| --- | --- | --- | --- | --- | --- | --- | --- | --- | --- | --- | --- | --- | --- | --- |
| RVA/Bat-wt/CMR/BatLy17/2014/G30P[47] | G30 | P[47] | I22 | R15 | C15 | M14 | A25 | N15 | T17 | E22 | H17 | *Eidolon helvum* | *Pteropodidae* | F |
| **RVA/Bat-wt/GHA/K212/2009/G30P[47]** | G30 | P[47] | I22 | R15 | C15 | M14 | A25 | N15 | T17 | E22 | H17 | *Eidolon helvum* | *Pteropodidae* | F |
| RVA/Bat-wt/CMR/BatLy03/2014/G25P[43] | G25 | P[43] | I15 | R16 | C8 | M15 | A26 | N8 | T11 | E23 | H10 | *Eidolon helvum* | *Pteropodidae* | F |
| RVA/Bat-wt/SAU/KSA402/2012/G25P[43] | G25 | P[43] | I15 | R16 | C8 | M15 | A26 | N8 | T11 | E23 | H10 | *Eidolon helvum* | *Pteropodidae* | F |
