# Supplementary material for: At Least Seven Distinct Rotavirus Genotype Constellations in Bats with Evidence of Reassortment and Zoonotic Transmissions
Source: mBio. 2021 Jan 19;12(1):e02755-20. doi: 10.1128/mBio.02755-20 (PMC7845630; doi:10.1128/mBio.02755-20)
Supplement: FIG S2 [file mBio.02755-20-sf002.docx]

**Figure S2.** Heatmap of pairwise nucleotide identities (NI) of the unusual RVA strains: RVA/Horse-wt/ARG/E3198/2008/G3P[3] (a), RVA/Simian-tc/ZAF/SA11-H96/1958/G3P[2] (b), RVA/Human-tc/KEN/B10/1987/G3P[2] (c), RVA/Human-wt/SUR/2014735512/2013/G20P[28] (d). Grey colour indicates the nucleotide identities below 0.6 or lack of sequence information for the compared strain
