# Supplementary material for: At Least Seven Distinct Rotavirus Genotype Constellations in Bats with Evidence of Reassortment and Zoonotic Transmissions
Source: mBio. 2021 Jan 19;12(1):e02755-20. doi: 10.1128/mBio.02755-20 (PMC7845630; doi:10.1128/mBio.02755-20)
Supplement: TABLE S5 [file mBio.02755-20-st005.docx]

**Table S5**. The Genbank accession numbers of the reference RVA strains used in the study

| **Strains** | **VP1** | **VP2** | **VP3** | **VP4** | **NSP1** | **VP6** | **NSP3** | **VP7** | **NSP2** | **NSP4** | **NSP5** |
| --- | --- | --- | --- | --- | --- | --- | --- | --- | --- | --- | --- |
| RVA/Alpaca-tc/PER/SA44/2014/G3P40 |  |  |  | KT935478 |  |  |  |  |  |  |  |
| RVA/Alpaca-wt/PER/356/2010/G3P14 |  |  |  | KT878993.1 |  |  |  |  |  |  |  |
| RVA/Alpaca-wt/PER/Alp11B/2010/G35P50 |  |  |  | KY971955.1 |  |  |  | KY971977.1 |  |  |  |
| RVA/Bat-wt/KEN/322/Kwale/2015/G3P10 | MH285826.1 | MH285827.1 | MH285828.1 | MH285829.1 | MH285830.1 | MH285831.1 | MH285832.1 | MH285834.1 | MH285833.1 | MH285835.1 | MH285836.1 |
| RVA/Bat-wt/KEN/BATp39/2015/G36P51 | MH285837.1 | MH285838.1 | MH285839.1 | MH285840.1 | MH285841.1 | MH285842.1 | MH285843.1 | MH285845.1 | MH285844.1 | MH285846.1 | MH285847.1 |
| RVA/Bat-wt/SAU/KSA402/2012/G25P43 | KX420939.1 | KX420940.1 | KX420941.1 | KX420942.1 | KX420943.1 | KX420944.1 | KX420947.1 | KX420946.1 | KX420945.1 | KX420949.1 | KX420948.1 |
| RVA/Bat-tc/CHN/MSLH14/2012/G3P3 | KC960619.1 | KC960620.1 | KC960621.1 | KC960622.1 | KC960625.1 | KC960623.1 | KC960627.1 | KC960626.1 | KC960624.1 | KC960628.1 | KC960629.1 |
| RVA/Bat-tc/CHN/MYAS33/2013/G3P10 | KJ020891.1 | KJ020892.1 | KJ020893.1 | KF649187.1 | KJ020887.1 | KJ020894.1 | KJ020889.1 | KF649188.1 | KJ020888.1 | KJ020890.1 | KF649186.1 |
| RVA/Bat-wt/BRA/3081/2013/G20Px |  |  |  | KR106166.1 |  |  | KR106164.1 | KR106163.1 |  |  | KR106165.1 |
| RVA/Bat-wt/BRA/4754/2013/G3P3 |  |  |  | KR106161.1 |  |  |  | KR106162.1 |  | KR106159.1 | KR106160.1 |
| RVA/Bat-wt/CHN/BSTM70/2015/G3P3 | KX814924.1 | KX814925.1 | KX814926.1 | KX814922.1 | KX814927.1 | KX814923.1 | KX814928.1 | KX814929.1 | KX814921.1 | KX814930.1 | KX814931.1 |
| RVA/Bat-wt/CHN/GLRL1/2005/G33P48 | KX814935.1 | KX814936.1 | KX814937.1 | KX814933.1 |  | KX814934.1 | KX814939.1 | KX814932.1 | KX814938.1 | KX814941.1 | KX814940.1 |
| RVA/Bat-wt/CHN/LZHP2/2015/G3P3 | KX814945.1 | KX814946.1 | KX814947.1 | KX814943.1 | KX814948.1 | KX814944.1 | KX814950.1 | KX814942.1 | KX814949.1 | KX814951.1 | KX814952.1 |
| RVA/Bat-wt/CHN/YSSK5/2015/G3P3 | KX814956.1 | KX814957.1 | KX814958.1 | KX814954.1 | KX814959.1 | KX814955.1 | KX814961.1 | KX814953.1 | KX814960.1 | KX814962.1 | KX814963.1 |
| RVA/Bat-wt/CMR/BatLi08/2014/G31P42 | KX268765.1 | KX268766.1 | KX268767.1 | KX268768.1 | KX268771.1 | KX268769.1 | KX268773.1 | KX268770.1 | KX268772.1 | KX268774.1 | KX268775.1 |
| RVA/Bat-wt/CMR/BatLi09/2014/G30P42 | KX268754.1 | KX268755.1 | KX268756.1 | KX268757.1 | KX268760.1 | KX268758.1 | KX268762.1 | KX268759.1 | KX268761.1 | KX268763.1 | KX268764.1 |
| RVA/Bat-wt/CMR/BatLi10/2014/G30P42 | KX268743.1 | KX268744.1 | KX268745.1 | KX268746.1 | KX268749.1 | KX268747.1 | KX268751.1 | KX268748.1 | KX268750.1 | KX268752.1 | KX268753.1 |
| RVA/Bat-wt/CMR/BatLy03/2014/G25P43 | KX268776.1 | KX268777.1 | KX268778.1 | KX268779.1 | KX268782.1 | KX268780.1 | KX268784.1 | KX268781.1 | KX268783.1 | KX268785.1 | KX268786.2 |
| RVA/Bat-wt/CMR/BatLy17/2014/G30P47 | KX268787.1 | KX268789.1 | KX268790.1 | KX268788.1 | KX268793.1 | KX268791.1 | KX268795.1 | KX268792.1 | KX268794.1 | KX268796.1 | KX268797.1 |
| RVA/Bat-wt/KEN/KE4852/2007/G25P6 |  | GU983673.1 |  | GU983674.1 |  | GU983675.1 | GU983678.1 | GU983676.1 | GU983677.1 | GU983679.1 | GU983680.1 |
| RVA/Bat-wt/ZMB/LUS12-14/2012/G3P3 | LC158119.1 | LC158120.1 | LC158121.1 | LC158117.1 | LC158122.1 | LC158118.1 | LC158116.1 | LC158123.1 | LC158124.1 | LC158125.1 | LC158126.1 |
| RVA/Bat-wt/ZMB/ZFB14-126/2014/GxPx |  |  |  |  |  | LC277165.1 | LC277163.1 |  | LC277162.1 | LC277164.1 |  |
| RVA/Bat-wt/ZMB/ZFB14-135/2014/G31Px | LC277168.1 |  |  |  |  | LC277169.1 | LC277167.1 | LC277170.1 |  |  |  |
| RVA/Bat-wt/ZMB/ZFB14-52/2014/G31Px |  |  |  |  |  | LC277160.1 | LC277159.1 | LC277161.1 |  |  |  |
| RVA/Camel/KUW/s21/2010/G10P15 |  |  |  | JX968470.2 |  |  |  |  |  |  |  |
| RVA/Camel-wt/SDN/MRC-DPRU447/2009/G8P1 |  |  |  |  | KC257086.1 |  |  |  |  | KC257089.1 |  |
| RVA/Chicken-tc/DEU/02V0002G3/2002/G19P30 | FJ169853.1 | FJ169854.1 | FJ169855.1 | FJ169856.1 | FJ169857.1 | FJ169858.1 | FJ169859.1 | FJ169861.1 | FJ169860.1 | FJ169862.1 | FJ169863.1 |
| RVA/Chicken-tc/GBR/Ch-1/197x/G19P17 |  |  |  |  |  | D82970.1 |  | AB080738.1 |  |  |  |
| RVA/CommonGull-wt/JPN/Ho374/2013/G28P39 | LC088218.1 | LC088219.1 | LC088220.1 | LC088221.1 | LC088224.1 | LC088222.1 | LC088225.1 | LC088226.1 | LC088223.1 | LC088227.1 | LC088228.1 |
| RVA/Cow-tc/GBR/PP-1/1976/G3P7 |  |  |  |  |  |  |  | AF427124.1 |  | AF427521.1 |  |
| RVA/Bovine-tc/USA/UK/1984/G6P5 |  |  |  | JF693051.1 |  |  |  |  |  |  |  |
| RVA/Cow-tc/IND/Hg18/1995/G15P21 |  |  |  | AF237665.1 |  |  |  | AF237666.1 |  |  |  |
| RVA/Cow-tc/JPN/Dai-10/2007/G24P33 | AB573070.1 | AB573071.1 | AB573072.1 | AB513836.1 | AB573074.1 | AB573073.1 | AB573075.1 | AB573076.1 | AB513837.1 | AB573077.1 | AB573078.1 |
| RVA/Cow-tc/THA/A5-13/G8P1 |  |  |  | LC133528.1 |  |  |  |  |  |  |  |
| RVA/Cow-tc/USA/B223/G10P11 |  |  |  | LC133550.1 |  |  |  |  |  |  |  |
| RVA/Cow-tc/USA/NCDV/1971/G6P1 | DQ870493.1 | DQ870494.1 |  |  |  |  |  |  |  |  |  |
| RVA/Cow-tc/USA/WC3/1981/G6P5 | EF560615.1 | EF560616.1 | EF560617.1 |  | EF990699.1 |  | EF990701.1 |  | EF990700.1 |  | EF990702.1 |
| RVA/Cow-wt/JPN/Azuk-1/2006/G21P29 |  |  |  | LC553631.1 |  |  | LC553636.1 |  |  |  |  |
| RVA/Dog-wt/HUN/135/2012/G3P3 | KJ875791.1 | KJ875792.1 | KJ875793.1 | KJ875794.1 | KJ875797.1 | KJ875795.1 | KJ875799.1 | KJ875796.1 | KJ875798.1 | KJ875800.1 | KJ875801.1 |
| RVA/Dog-tc/ITA/RV198-95/1995/G3P3 | HQ661134.1 | HQ661135.1 | HQ661136.1 | HQ661137.1 | HQ661140.1 | HQ661138.1 | HQ661142.1 | HQ661139.1 | HQ661141.1 | HQ661143.1 | HQ661144.1 |
| RVA/Guanaco-wt/ARG/Chubut/1999/G8P14 | FJ347100.1 | FJ347101.1 | FJ347102.1 | FJ347103.1 | FJ347106.1 | FJ347104.1 | FJ347108.1 | FJ347105.1 | FJ347107.1 | FJ347109.1 | FJ347110.1 |
| RVA/Horse-tc/USA/FI14/1981/G3P12 |  |  |  |  | KM454487.1 |  |  |  |  |  |  |
| RVA/Horse-tc/GBR/H-2/1976/G3P12 |  |  |  | KM454495.1 |  |  |  |  |  |  |  |
| RVA/Horse-tc/GBR/L338/1991/G13P18 | JF712555.1 | JF712556.1 | JF712557.1 | JF712558.1 | JF712561.1 | JF712559.1 | JF712560.1 | JF712562.1 | JF712563.1 | JF712564.1 | JF712565.1 |
| RVA/Horse-tc/USA/FI23/1981/G14P12 |  |  |  |  |  |  |  | KM454508.1 |  |  |  |
| RVA/Horse-wt/ARG/E30/1993/G3P12 |  |  |  |  |  |  |  |  |  |  | JF712576.1 |
| RVA/Horse-wt/ARG/E3198/2008/G3P3 | JX036365.1 | JX036366.1 | JX036367.1 | JX036368.1 | JX036371.1 | JX036369.1 | JX036373.1 | JX036370.1 | JX036372.1 | JX036374.1 | JX036375.1 |
| RVA/Human-wt/ITA/ME848/2012/G12P8 | KR632623.1 | KR632624.1 | KR632625.1 | KR632621.1 | KR632626.1 | KR632622.1 | KR632628.1 | KR632620.1 | KR632627.1 | KR632629.1 | KR632630.1 |
| RVA/Human/CHN/ZTR-5/XXXX/G3P2 | JF896465.1 | JF896466.1 | JF896467.1 | JF896468.1 | JF896471.1 | JF896469.1 | JF896473.1 | JF896470.1 | JF896472.1 | JF896474.1 | JF896475.1 |
| RVA/Human-tc/KEN/B10/1987/G3P2 | HM627553.1 | HM627554.1 | HM627555.1 | HM627556.1 | HM627559.1 | HM627557.1 | HM627561.1 | HM627558.1 | HM627560.1 | HM627562.1 | HM627563.1 |
| RVA/Human-tc/CHN/L621/2006/G3P9 | JX946159.1 | JX946160.1 | JX946161.1 | EU708574.1 | JX946163.1 | JX946162.1 | JX946165.1 | EU708588.1 | JX946164.1 | JX946166.1 | JX946167.1 |
| RVA/Human-tc/GBR/A64/1987/G10P1114 |  |  |  |  |  |  |  | EF672567.1 |  |  |  |
| RVA/Human-tc/GBR/ST3/1975/G4P2 |  |  |  |  |  |  |  | EF672616.1 |  |  |  |
| RVA/Human-tc/IND/116E/1985/G9P11 |  |  |  | FJ361204.1 |  |  |  |  |  |  |  |
| RVA/Human-tc/IND/69M/1980/G8P10 |  |  |  | M60600.1 |  |  |  | EF672560.1 |  |  |  |
| RVA/Human-tc/JPN/AU-1/1982/G3P9 | DQ490533.1 | DQ490536.1 | DQ490537.1 | D10970 | D45244 | DQ490538.1 | DQ490535.1 | D86271.1 | DQ490534.1 |  | AB008656 |
| RVA/Human-tc/THA/Mc323/1989/G9P19 |  |  |  | D38052.1 |  |  |  |  |  |  |  |
| RVA/Human-tc/THA/T152/1998/G12P9 |  |  |  |  |  |  |  |  |  |  | DQ146706.1 |
| RVA/Human-tc/USA/DS-1/1976/G2P1B4 | HQ650116.1 | HQ650117.1 | HQ650118.1 | HQ650119.1 | HQ650120.1 | HQ650121.1 | HQ650122.1 | HQ650124.1 | HQ650123.1 | HQ650125.1 | HQ650126.1 |
| RVA/Human-tc/USA/Wa/1974/G1P1A8 | KT694939.1 | KT694940.1 | KT694941.1 | KT694942.1 | KT694945.1 | KT694943.1 | KT694947.1 | KT694944.1 | KT694946.1 | KT694948.1 | KT694949.1 |
| RVA/Human-tc/USA/WI61/1983/G9P1A8 |  |  |  |  |  |  |  | LC482504.1 |  |  |  |
| RVA/Human-wt/BEL/B4106/2000/G3P14 |  |  |  |  |  |  |  |  |  | AY740732.1 |  |
| RVA/Human-wt/BEL/BEF06018/2014/G29P41 |  |  |  | KU128895.1 |  |  |  |  |  |  |  |
| RVA/Human-wt/BGD/Dhaka6/2001/G11P25 |  |  |  | AY773004.2 |  |  |  |  |  |  |  |
| RVA/Human-wt/BRA/QUI-35-F5/2010/G3P9 |  |  |  |  | KF185099.1 | KF185107.1 |  |  |  |  |  |
| RVA/Human-wt/CHN/E2451/2011/G3P9 | JX946168.1 | JX946169.1 | JX946170.1 | JX946171.1 | JX946174.1 | JX946172.1 | JX946176.1 | JX946173.1 | JX946175.1 | JX946177.1 | JX946178.1 |
| RVA/Human-wt/ECU/Ecu534/2006/G20P28 |  |  |  | EU805773.1 |  | EU805774.2 |  | EU805775.1 |  |  |  |
| RVA/Human-wt/HUN/Hun5/1997/G6P14 |  |  |  |  | EF554110.1 |  |  |  |  |  |  |
| RVA/Human-wt/NPL/KTM368/2004/G11P25 |  |  |  |  |  | GU199496.1 |  |  |  |  |  |
| RVA/Human-wt/SUR/2014735512/2013/G20P28 | KX257410.1 | KX257409.1 | KX257408.1 | KX257407.1 | KX257415.1 | KX257406.1 | KX257414.1 | KX257413.1 | KX257405.1 | KX257412.1 | KX257411.1 |
| RVA/Human-wt/THA/CMH222/2001/G3P3 |  |  |  | DQ288661.1 |  | DQ288659.1 |  | AY707792.1 |  | DQ288660.1 |  |
| RVA/Human-wt/US/09US7118/2009/G3P24 | KF541281.1 | KF541282.1 | KF541283.1 | KF541284.1 | KF541287.1 | KF541285.1 | KF541288.1 | KF541289.1 | KF541286.1 | KF541290.1 | KF541291.1 |
| RVA/Alpaca/PER/ALRVA-Kayra/3386/2010/G3Px |  |  |  |  |  |  |  | KT250942.1 |  |  |  |
| RVA/Mouse-tc/UK/EHP/1981/G16P20 |  |  |  | U08424.1 |  |  |  |  |  |  |  |
| RVA/Mouse-tc/USA/ETD_822/2007/G16P16 | GQ479947.1 | GQ479948.1 | GQ479949.1 | GQ479950.1 |  |  | GQ479953.1 |  | GQ479954.1 |  | GQ479957.1 |
| RVA/Mouse-tc/USA/EW/XXXX/G16P16 |  |  |  | U08429.1 | U08428.1 | U36474.1 |  | U08430.1 |  | U96335.1 |  |
| RVA/Pheasant-tc/GER/10V0112H5/2010/G23P37 |  |  |  | JX204814.1 |  |  |  |  |  |  |  |
| RVA/Pheasant-wt/HUN/Phea14246/2008/G23Px |  |  |  |  |  |  |  | FN393054.1 |  |  |  |
| RVA/Pigeon-tc/JPN/PO-13/1983/G18P17 | AB009629.2 | AB009630.2 | AB009631.2 | AB009632.2 | AB009633.2 | D16329.2 | AB009626.2 | D82979.2 | AB009625.2 | AB009627.1 | AB009628.1 |
| RVA/Pig-tc/USA/OSU/1975/G5P7 |  |  |  | KR052770.1 |  |  |  | KR052772.1 |  |  |  |
| RVA/Pig-wt/XXX/A46/XXXX/GxP13 |  |  |  | AY050274.1 |  |  |  |  |  |  |  |
| RVA/Pig-wt/BGD/2014016006/G9P49 |  |  |  | KY905314.1 |  |  |  |  |  |  |  |
| RVA/Pig-wt/CAN/CE-M-06-0003/2006/G2P27 |  |  |  |  |  | GU183245.1 |  |  |  |  |  |
| RVA/Pig-wt/CHN/NMTL/2008/G9P23 |  |  |  | JF781161.1 |  |  |  |  |  |  |  |
| RVA/Pig-wt/IRL/61-07-ire/2007/G2P32 |  |  |  | FJ492835.1 |  |  |  |  |  |  |  |
| RVA/Pig-wt/JPN/FGP51/2009/G4P34 |  |  |  | AB571047.1 |  |  |  |  |  |  |  |
| RVA/Pig-wt/JPN/TJ41/2010/G26Px |  |  |  |  |  |  |  | AB605258.1 |  |  |  |
| RVA/Pig-wt/THA/CMP034/2000/G2P27 |  |  |  | DQ534016.2 |  |  |  |  |  |  |  |
| RVA/Rabbit-wt/ITA/160/01/2001/G3P22 |  |  |  | AF526374.1 |  |  |  |  |  |  |  |
| RVA/Raccoon-tc/JPN/Rac-311/2011/G34P17 |  | LC208546.1 | LC208547.1 |  | LC208551.1 | LC208549.1 | LC208553.1 | LC208550.1 | LC208552.1 |  |  |
| RVA/Rat/CHN/RA116/2013/G3P45 |  |  |  | KU243630.1 |  |  |  |  |  |  | LC208555.1 |
| RVA/Rat-wt/GER/KS-11-573/2011/G3P3 | KJ879448.1 | KJ879449.1 | KJ879450.1 |  | KJ879454.1 | KJ879452.1 | KJ879456.1 |  |  | KJ879457.1 | KJ879458.1 |
| RVA/Rat-wt/ITA/Rat14/2015/G3P3 |  |  |  |  |  |  |  |  | KX398370.1 |  |  |
| RVA/Rhesus-tc/USA/TUCH/2002/G3P24 | EF583010.1 | EF583011.1 | EF583012.1 | FJ816611.1 | FJ816612.1 | EF583013.1 | FJ816613.1 | FJ816615.1 | FJ816614.1 | FJ816616.1 | FJ816617.1 |
| RVA/Sheep-wt/CHN/Lp14/xxxx/G10P15 |  |  |  | L11599.1 |  | L11595.1 |  |  |  |  |  |
| RVA/Shrew-wt/CHN/LW9/2013/G28P46 | KU243542.1 | KU243571.1 | KU243598.1 | KU243621.1 | KU243377.1 | KU243658.1 | KU243457.1 | KU243688.1 | KU243430.1 | KU243476.1 | KU243505.2 |
| RVA/Simian-tc/USA/RRV/1975/G3P3 | EU636924.1 | EU636925.1 | EU636926.1 | EU636927.1 | EU636928.1 | EU636929.1 | EU636930.1 | EU636932.1 | EU636931.1 | EU636933.1 | EU636934.1 |
| RVA/Simian-tc/USA/SA11g4O-5N/G3P1 |  |  |  | EU636934.1 |  |  |  |  |  |  |  |
| RVA/Simian-tc/ZAF/SA11-H96/1958/G3P2 | DQ838640.2 | DQ838635.2 | DQ838645.2 | DQ841262.2 | DQ838599.2 | DQ838650.2 | DQ838610.2 | DQ838620.2 | DQ838615.2 | DQ838625.2 | DQ838630.2 |
| RVA/SugarGlider-tc/JPN/SG385/2012/G27P36 | AB971760.1 | AB971761.1 | AB971762.1 | AB971763.1 | AB971766.1 | AB971764.1 | AB971768.1 | AB971765.1 | AB971767.1 | AB971769.1 | AB971770.1 |
| RVA/Turkey-tc/DEU/03V0002E10/2003/G22P35 |  |  |  | JX204825.1 |  |  |  | JX204830.1 |  |  |  |
| RVA/Turkey-tc/IRL/Ty-1/1979/G17P38 |  |  |  | LC088110.1 |  |  |  | S58166.1 |  |  |  |
| RVA/Turkey-tc/IRL/Ty-3/1979/G7P35 |  |  |  |  |  |  |  | LC088119.1 |  |  |  |
| RVA/VelvetScoter-tc/JPN/RK1/1989/G18P17 |  |  |  |  | LC088102.1 |  |  |  |  |  |  |
| RVA/Vicuna-wt/ARG/C75/2010/G8P14 |  |  |  |  |  |  |  |  |  | JX070055.1 |  |
